# Serial stenosis assessment—can we rely on invasive coronary physiology

**DOI:** 10.3389/fcvm.2023.1172906

**Published:** 2023-05-02

**Authors:** Ivan Ilic, Stefan Timcic, Natalija Odanovic, Petar Otasevic, Carlos Collet

**Affiliations:** ^1^ Institute for Cardiovascular Diseases Dedinje, Belgrade, Serbia; ^2^ Medical School, University of Belgrade, Belgrade, Serbia; ^3^ Cardiovascular Center Aalst, OLV Clinic, Aalst, Belgium

**Keywords:** coronary artery stenosis, fractional flow reserve (FFR), pullback pressure gradient, IFR, serial stenosis

## Abstract

Atherosclerosis is a widespread disease affecting coronary arteries. Diffuse atherosclerotic disease affects the whole vessel, posing difficulties in determining lesion significance by angiography. Research has confirmed that revascularization guided by invasive coronary physiology indices improves patients' prognosis and quality of life. Serial lesions can be a diagnostic challenge because the measurement of functional stenosis significance using invasive physiology is influenced by a complex interplay of factors. The use of fractional flow reserve (FFR) pullback provides a trans-stenotic pressure gradient (*Δ*P) for each of the lesions. The strategy of treating the lesion with greater *Δ*P first and then reevaluating another lesion has been advocated. Similarly, non-hyperemic indices can be used to assess the contribution of each stenosis and predict the effect of lesion treatment on physiology indices. Pullback pressure gradient (PPG) integrates physiological variables of coronary pressure along the epicardial vessel and characteristics of discrete and diffuse coronary stenoses into a quantitative index that can be used to guide revascularization. We proposed an algorithm that integrates FFR pullbacks and calculates PPG to determine individual lesion importance and to guide intervention. Computer modeling of the coronaries and the use of non-invasive FFR measurement together with mathematical algorithms for fluid dynamics can make predictions of lesion significance in serial stenoses easier and provide practical solutions for treatment. All these strategies need to be validated before widespread clinical use.

## Introduction

Atherosclerosis is a widespread disease of the cardiovascular system. It affects the entire epicardial coronary artery tree. Assessment of the lesion significance can be challenging, especially in diffuse atherosclerotic disease. From the treatment perspective, multiple stenoses pose difficulties in identifying the most significant lesion to treat with the goal of achieving complete revascularization and avoiding long segment stenting, which can be prone to restenosis and thrombosis (1, 2).

**Figure 1:**
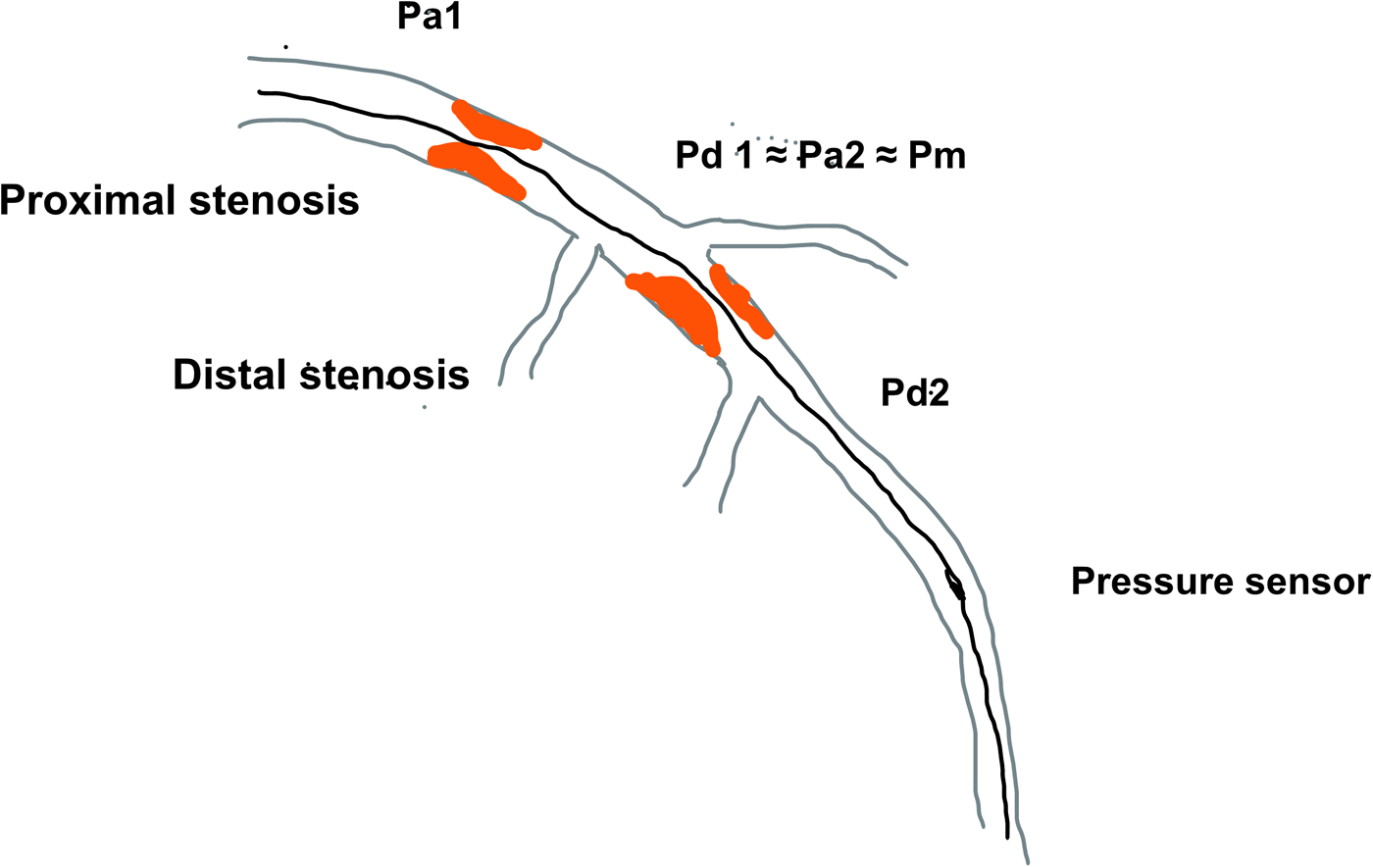
The schematic diagram of serial coronary artery stenoses and pressures measured using pressure wire Pa—proximal arterial pressure; Pd, distal arterial pressure; Pm, medial (between lesion) pressure.

Large clinical trials have confirmed that revascularization based on coronary physiology measurement with indices like fractional flow reserve (FFR) and resting (non-hyperemic) (NHI) indices like instantaneous wave-free ratio (iFR), resting full cycle ratio (RFR), and others improve patients' prognosis and quality of life (3–5). Although the assessment of lesion severity has been greatly simplified by using pressure measurement during maximal hyperemia as a surrogate of flow measurement, there are still procedural pitfalls that can compromise the reliability of the obtained results (6) (Figure 1).

## The hemodynamics of serial stenoses

The effect of serial stenosis on pressure changes in the coronary arteries is influenced by a complex interplay of several factors. Since the pivotal work of L. Gould, it has been stated that pressure change (***Δ*P**) across the lesion is affected by frictional and separation coefficients in a simplified equation that includes flow - ***Δ*P = fQ + sQ^2^,** where **Q** stands for coronary flow and **f** is the friction coefficient presenting the effect of viscosity of the blood as a non-Newtonian fluid and **s** is the separation coefficient representing the effects of vessel lumen configuration and geometry of stenosis (6–8). This simplified equation encompasses the influence of several lesion characteristics on pressure changes embedded in Poiseuille's law and Bernoulli equation. A drop in pressure across the lesion is thus influenced by luminal narrowing or a degree of stenosis, lesion length, and flow conditions. After successful PCI, there is an important change in perfusion pressure and microvascular resistance that would certainly affect FFR measurement in the remaining lesion.

**Figure 2:**
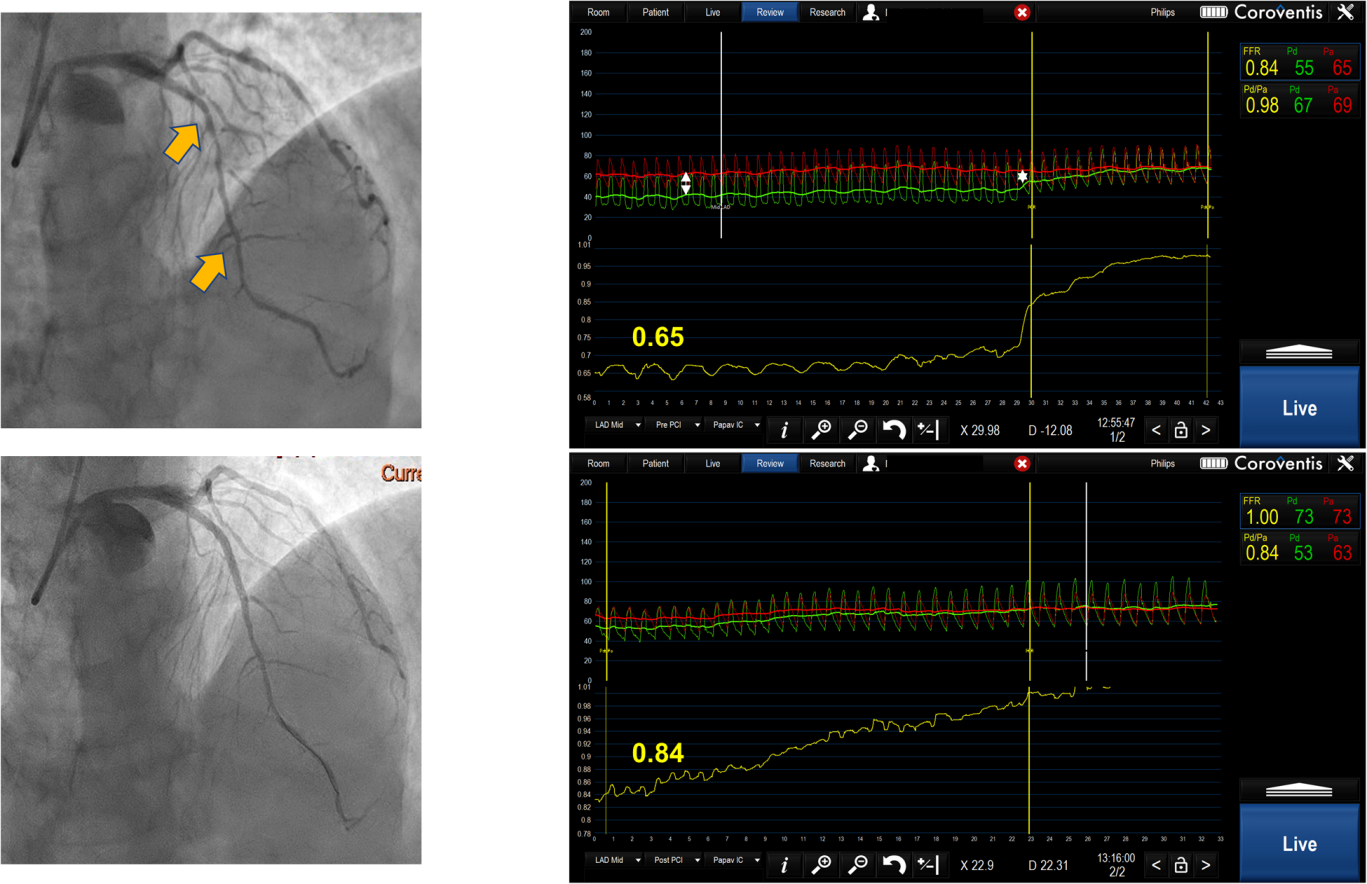
Serial lesion assessment by pullback FFR measurement using Coroventis CoroFlow cardiovascular software (Coroventis AB, Uppsala, Sweden), followed by PCI of the lesion with greatest *Δ*P and retesting after stent implantation leading to the deferral of further intervention due to an increase in FFR.

This effect of “removing” stenosis after PCI and subsequent changes in epicardial resistance and *Δ*P plays an important role in coronaries where multiple stenoses are present (9). If there are consecutive lesions in a single coronary artery, the estimation of FFR is cumulatively affected by the pressure drop at each lesion. The measurement of FFR with a pressure sensor positioned distal to both lesions would measure pressure drop across both lesions (*Δ*P) and estimate lesion significance throughout the entire coronary artery. In serial stenoses, for clarity, the proximal lesion is defined as lesion 1 with its proximal (Pa1) pressure and distal (Pd1) and the distal lesion would be termed lesion 2 (Pa2, Pd2). In this case, the distal pressure for lesion 1 (Pd1) would be similar to lesion 2′s proximal pressure (Pa2) which is further termed medial pressure (Pm), so that accurate measurement of the true FFR or NHI of each lesion cannot be done by placing the pressure sensor after lesion 1 and then after lesion 2. The reason for this is that pressure drop across lesion 1 does not reflect the myocardial ischemia in the territory of the supplying artery since the distal pressure for this lesion is not the pressure at the level of the distal artery, but it is significantly higher due to another stenosis distally. The same is true for distal lesions because their proximal pressure is not an aortic pressure but the distal pressure of the proximal lesion which has already dropped due to proximal stenosis (Figures 1, 2).

The founders of FFR De Bruyne and Pijls developed an animal model to measure FFR of each separate stenosis in a series of two artificially created stenoses of a canine coronary artery. The model included the measurement of distal wedge pressure (Pw) by inflating a balloon in a coronary artery and measuring the distal pressure, which represents wedge pressure. This calculation showed a good correlation with the true values of FFR for separate stenoses (10). This model was further validated in humans by the same authors in a relatively small cohort of patients where the predicted values of FFR for each lesion had a high correlation with the true values of FFR after one of the lesions was treated with stent implantation. The discrepancy between measured FFR and true FFR prior to PCI was approximately 15% for proximal lesion, while it was approximately 10% for distal stenosis, demonstrating the greater dependence of proximal stenosis to the distal one when measuring FFR. This method requires complex mathematical calculation and measurement of Pw which usually means that one lesion must be treated at least with a balloon angioplasty prior to the evaluation of stenosis significance. Also, this model excludes serial stenoses with significant side branches between them. Finally, after successfully treating one stenosis, the remaining pressure gradient and pathological FFR value might not be caused by adjacent stenosis but by diffuse atherosclerotic disease or visually non-significant stenosis proximal to the lesions being evaluated (10, 11).

## The methods to evaluate serial stenoses

There have been multiple attempts to find a reliable method to evaluate the significance of serial lesions in a single coronary artery. There is a consensus that using the visual estimation of each stenosis significance and/or measuring FFR by placing the pressure sensor between the lesions and assuming that the pressure measured represents Pd for proximal lesion cannot be accepted as valid.

FFR pullback can give a trans-stenotic pressure gradient for each lesion constituting tandem lesions. Treating the lesion with the greatest *Δ*P first and then reevaluating the other lesion is a reasonable approach (Figure 2). This strategy has demonstrated benefits in relatively small registries (12). When treating the lesion with greater *Δ*P, one might end up stenting long segments of diffusely diseased arteries, which may be revascularized more appropriately by surgical grafting. Further, serial lesion assessment using FFR pullback can lead to an underestimation of each stenosis significance, which may cause misinterpretation in selecting a lesion with a greater *Δ*P. Also, this technique requires stable hyperemia with intravenous adenosine or intracoronary papaverine with a fixed guiding catheter positioned against the ostium of the artery, which sometimes may be difficult to achieve. Finally, performing manual pullback cannot guarantee steady pullback velocity, which can result in an artificial plateau that can be interpreted as a smaller degree of change in pressure gradient, resulting in stenosis underestimation.

**Figure 3:**
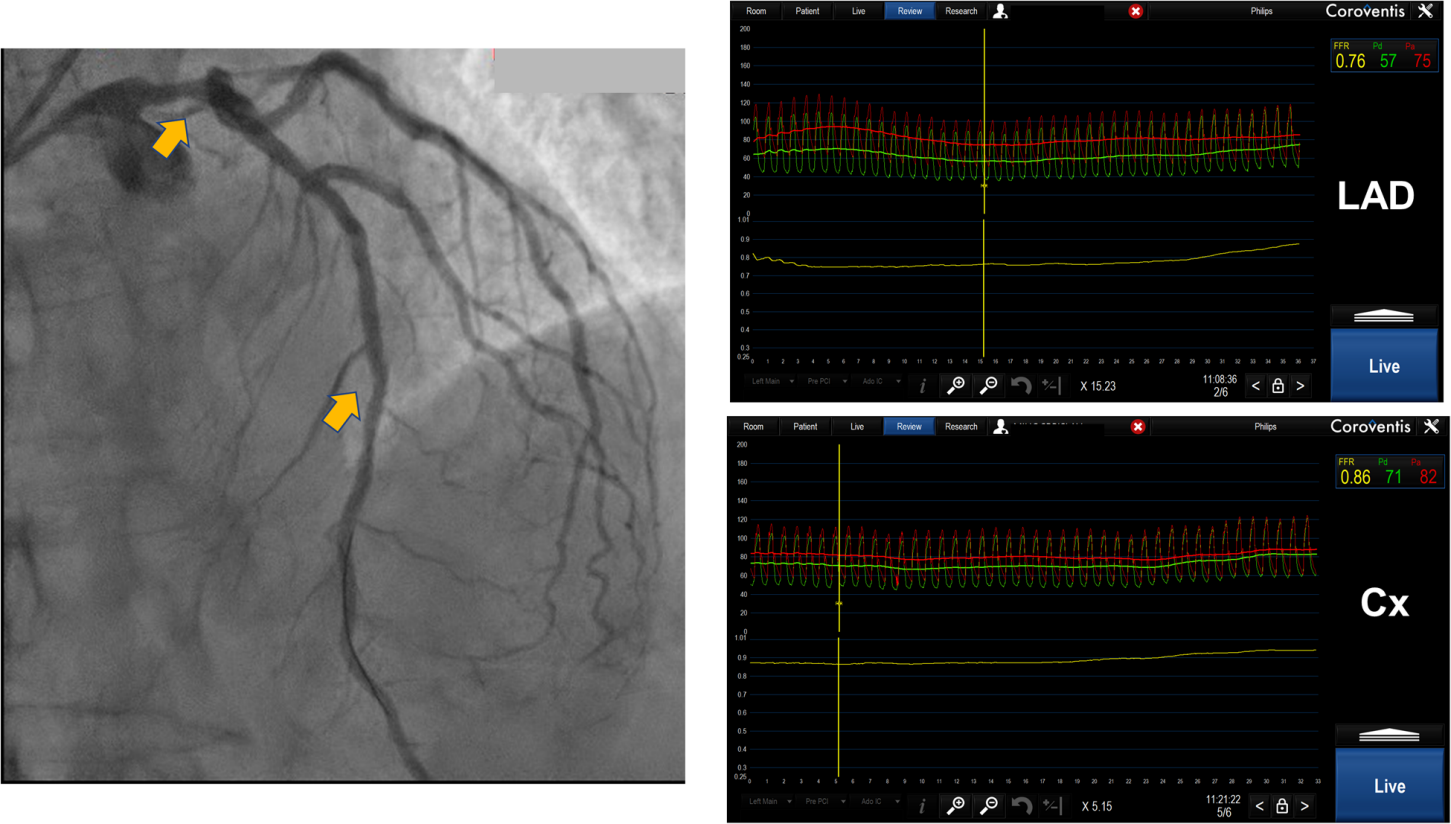
Distal left main lesion evaluation with downstream stenosis in LAD using Coroventis CoroFlow cardiovascular software (Coroventis AB, Uppsala, Sweden). Placing a pressure sensor in distal LAD FFR 0.76 was acquired while placing a sensor in distal Cx, free from disease, obtained FFR was 0,86. The lesion of distal LM was deemed insignificant.

When there is a disease-free distal branch that supplies a significant amount of myocardium, then performing FFR and hyperemic pullback from a diseased and disease-free branch may be a method to determine the significance of proximal stenosis. This has found use in bifurcation lesions with proximal main branch stenosis including left main (LM) stenosis that was associated with downstream stenosis in circumflex (Cx) and left anterior descending (LAD). Animal and human studies have shown that FFR in the presence of an LM stenosis and downstream disease when a pressure sensor is placed in the disease-free branch tends to underestimate the true value of FFR. This effect was more pronounced if the degree of stenosis is greater in the diseased branch. Knowing this, the operators have to be cautious when using FFR to evaluate the significance of LM stenosis in the presence of downstream stenosis because Cx usually supplies relatively smaller myocardial territory compared to LAD. We suggest that, in case of doubt, an invasive physiology investigation should be supplanted with a morphological investigation like intravascular ultrasound (IVUS) or optical coherence tomography (OCT) in order to determine the true hemodynamic significance of LM stenosis (13, 14), (Figure 3).

**Figure 4:**
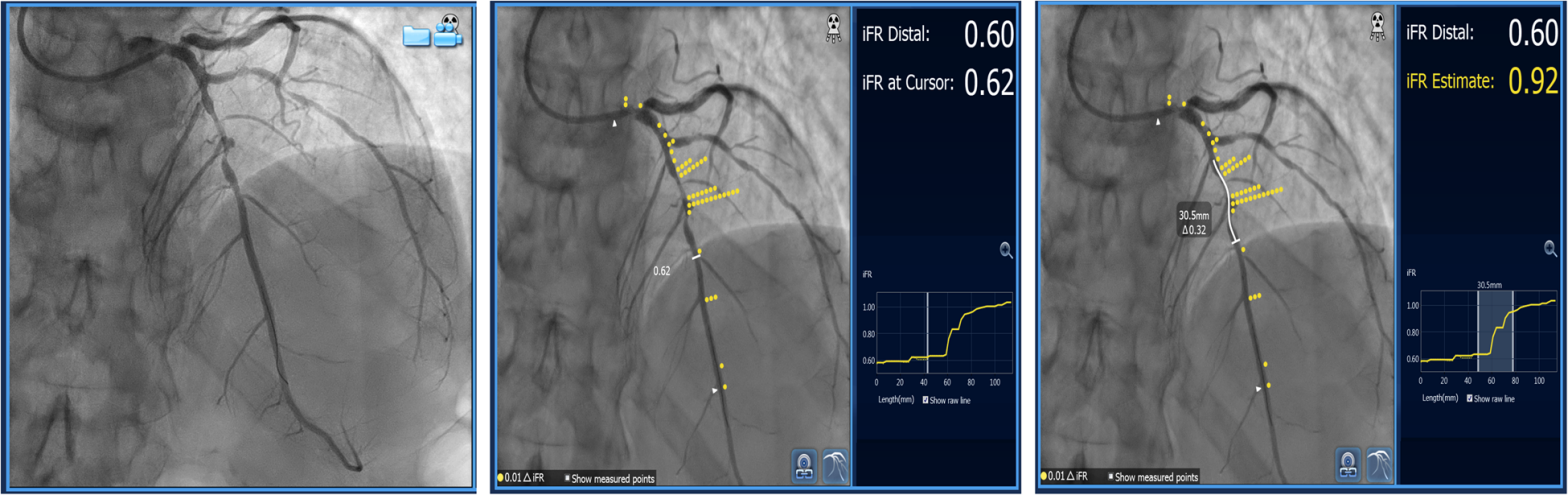
Serial lesion assessment using iFR and co-registration with “Sync Vision” software (Philips Healthcare, The Netherlands). The images show mid-LAD serial lesions assessed by iFR, and each yellow dot represents a 0.01 value in iFR Reading. The white-butted line in the right image demonstrates the potential effect of stent implantation on iFR value change (*Δ*iFR).

Resting or non-hyperemic indices (NHI) can be used to evaluate serial stenoses. Since they measure pressure differences in different parts of the myocardial contraction cycle, it has been speculated that they might be less affected by the interplay between serial lesions compared to FFR (6). They can be used in a similar manner as FFR by performing a pullback recording. Instantaneous wave-free ratio (iFR) (SyncVision, Philips, The Netherlands) uses co-registration with angiography and can predict each lesion's significance and the effect of PCI on iFR measurement afterward (Figure 4). It was demonstrated in 128 patients (134 diffuse and/or serial lesions) enrolled in the iFR GRADIENT registry that using iFR pullback and a prediction software changed the PCI strategy in almost 1/3 of the patients with an accurate prediction of post-PCI iFR values (15). However, this study was carried out for intermediate-severity lesions and the findings cannot be extrapolated to every serial stenosis regardless of its severity. Also, this method can be applied if the assumed Pw is close to 0 mmHg, and it does not take into account the potential effect of flow diversion if the side branches existed between the lesions. As previously stated, treating one lesion increases coronary flow and may affect iFR and other (NHI) measurements. Knowing this, it would be prudent to retest iFR after treating the lesion with the greatest trans-stenotic gradient and then decide on the treatment of another lesion. Another study by Warisawa et al., compared pullbacks by iFR, FFR, and hyperemic iFR in tandem lesions with the idea of comparing these modalities in determining lesion dominance. The dominant lesion had a greater trans-stenotic gradient. After defining iFR pullback assessment as the reference, they found that FFR-pullback assessment classified the cases differently in 22.7% of the patients (20/88) in the following manner: 23.9% (11/46) proximal predominant cases were re-classified as distal predominance, while 21.4% (9/42) distal predominant cases were re-classified as proximal predominance. Delta iFR and delta FFR did not have a strong correlation in either the proximal or distal lesions (15). The discordance between iFR and FFR was more pronounced in less severe lesions (less diameter stenosis and greater iFR values) while there was no difference regarding lesion length, vessel localization, and size (16). Another study by the same group demonstrated that the pattern of disease, focal vs. diffuse, also significantly influenced the discordance between iFR and FFR pullbacks. The pattern FFR positive/iFR negative was more frequent in focal disease, while FFR negative/iFR positive was seen more often in diffuse disease (17), (Figure 4). These findings warrant caution when interpreting resting indices data in focal disease and serial stenoses, demonstrating similar limitations as previously described in hyperemic FFR.

Pullback pressure gradient (PPG) can be an added value that can complement the lesion evaluation using FFR pullback. It integrates physiological variables of coronary flow and characteristics of discrete and diffuse coronary stenoses into a quantitative index on a scale from zero to one that can be used clinically to guide revascularization. It is defined by maximal delta FFR units over 20 mm of arterial length (or 20% of the pullback duration) as a ratio to total vessel *Δ*FFR, modified by an additional term for relative length of physiological disease to total vessel length. In a landmark study, Collet et al. analyzed FFR pullbacks in 85 vessels and found that the PPG index, as a continuous variable, with average values of 0.37 ± 0.07 (28vessels) indicated primarily diffuse disease, while a PPG of 0.77 ± 0.08 (29 vessels) indicated primarily focal disease, and 0.57 ± 0.05 (28 vessels) indicated mixed disease, concluding that more diffuse disease was present at lower PPG values and more focal at higher PPG values (18). In a study by Mizukami et al. that included 113 patients (116 vessels) and used FFR pullback and OCT to evaluate the result of PCI, the focal disease was defined as PPG > 0.73. The intervention in vessels with high PPG (focal disease and average PPG 0.80 ± 0.06) resulted in higher post-PCI FFR compared to low PPG (diffuse disease and average 0.58 ± 0.09), (0.91 ± 0.07 focal vs. 0.86 ± 0.05 diffuse group, *P *< 0.001), and greater stent area (6.3 ± 2.3 mm^2^ vs. 5.3 ± 1.8 mm^2^, *P *= 0.015). The authors stated that PPG significantly improved the capacity to predict optimal PCI results compared with an angiographic assessment of CAD patterns (area under the curve PPG 0.81 [95% CI, 0.73–0.88] vs. area under the curve angio 0.51 [95% CI, 0.42–0.60]; *P *< 0.001) (19). Regarding post-PCI angina, in a sub-study of TARGET—FFR (Trial of Angiography vs. pressure-Ratio-Guided Enhancement Techniques—Fractional Flow reserve), it was shown that patients with focal disease had larger increases in FFR after PCI (0.30 ± 0.14 vs. 0.19 ± 0.12; *P* < 0.001) and less angina assessed by the Seattle angina questionnaire (SAQ-7) [87.1 ± 20.3 vs. 75.6 ± 24.4; mean difference = 11.5 (95% CI: 2.8–20.3); *P* = 0.01] compared to patients with diffuse disease assessed by PPG. Residual angina was present in more than one-third of patients but was significantly less in those with focal disease (27.5% vs. 51.9%; *P* = 0.020) (20).

**Figure 5:**
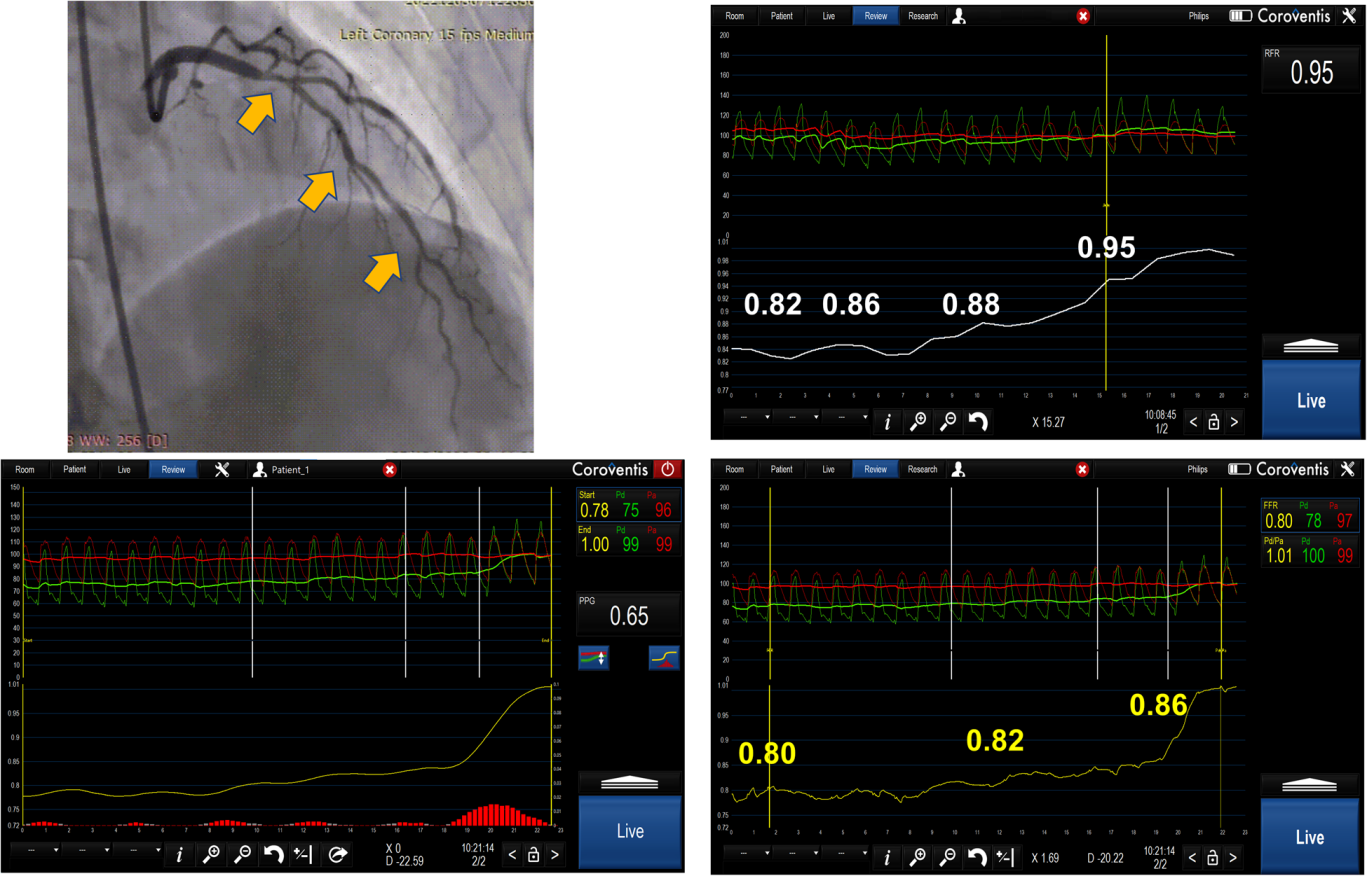
The image represents serial lesion assessment in a 39 year-old patient using resting full-cycle ratio (RFR) and FFR pullback with papaverine as the hyperemic agent. The obtained values were abnormal for RFR and normal for FFR. The possible reason for this could be that there was a diffuse atherosclerotic disease and pullback pressure gradient (PPG showing an intermediate value of 0.65) and the operator decided to defer revascularization due to a normal FFR value and diffuse disease in the left main and LAD.

The concept of PPG was applied to patients with serial stenoses, and it identified three distinct functional FFR phenotypes based on FFR curve pullbacks: one indicating diffuse disease with no step-up during pullback with low PPG values, second with FFR pullback curves with one step up, and finally an FFR pullback curve with two steps up and highest PPG values, indicating distinct stenoses that can be best served by PCI (21), (Figure 5). The use of PPG in guiding serial stenosis evaluation and PCI has not been systematically compared to FFR and NHI pullback, so it cannot replace already accepted methods in the algorithm of serial stenosis evaluation and treatment. In our opinion, PPG can be a new step in the evaluation that could help us to better identify the patients with true separate lesions that can be best served by PCI compared to the patients that have the diffuse disease and in whom, despite significant FFR or NHI values, optimal medical therapy might be a good solution.

## Future directions of research

Computational modeling to assess coronary physiology and the use of non-invasive imaging modalities to determine lesion significance using FFR and resting indices is rapidly developing to be able to tackle the issue of serial coronary artery stenoses (22). Three-dimensional printing that precisely reconstructs coronary artery geometry in addition to mathematical models that implement laws of fluid dynamics and interaction between adjacent stenoses could help to better estimate lesion significance and to guide intervention (23, 24). Novel coronary CT angiography-derived tool to assess the change in FFR after the revascularization of a single lesion in a coronary artery with serial lesions might be helpful in guiding PCI in this subset. A study has shown that using a CT-based software planning tool might better estimate the individual stenosis contribution in serial stenoses compared to FFR pullback and CT-derived FFR (25). These contemporary tools must be evaluated in large-scale clinical trials to gain an adequate appreciation and to be incorporated into the daily routine of an interventionalist.

## A practical approach for serial stenoses using invasive physiology

Both modalities NHIs and FFR can be used for serial stenoses evaluation in daily practice. The advantage of NHIs is that they measure pressure gradient in resting conditions based on resting flow, which is physiologically preserved until stenosis becomes critical and resting flow declines. Thus, the variations of NHIs for serial lesions are less pronounced after treating one of the lesions, which is especially true for iFR because it measures pressure gradient in diastole, which would be more prominent than if it was measured throughout the entire heart cycle (26). However, we think that an algorithm that integrates FFR and PPG would offer more advantage because it integrates two parameters, one that estimates pressure drop on discrete lesions (pullback FFR) and the other that evaluates the distribution of coronary artery disease in the entire artery (PPG), thus allowing the adequate treatment of focal lesions while avoiding treatment of diffuse disease that does not confer clinical benefits for the patient.

**Figure 6:**
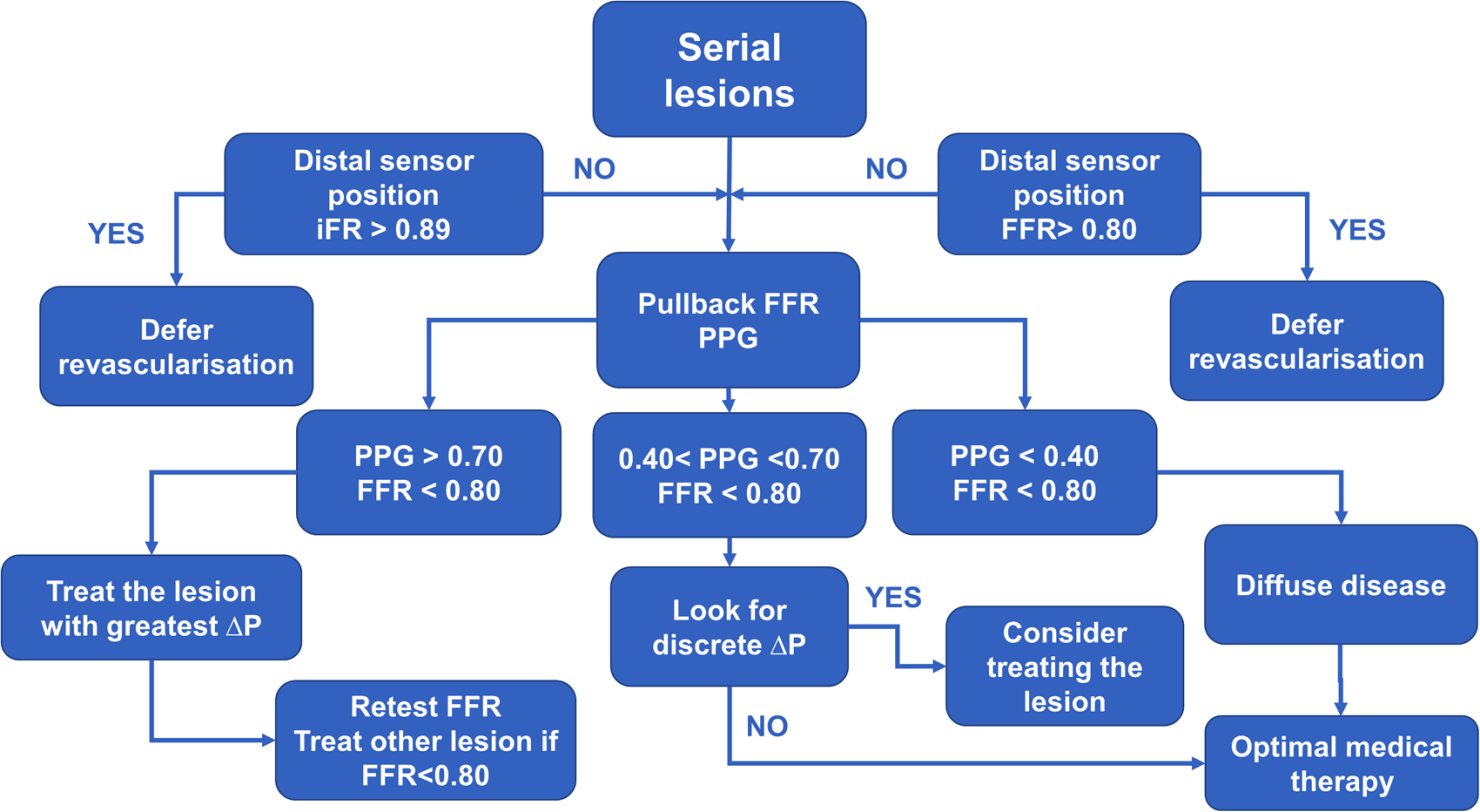
The diagram demonstrates practical approach to a patient with serial stenoses using invasive physiology (iFR – instantaneous wave free ratio, FFR – fractional flow reserve and PPG – pullback pressure gradient).

We propose an algorithm that incorporates FFR and PPG into serial stenoses evaluation. In a patient evaluated with invasive physiology, first, perform resting indices measurement with the pressure sensor placed distally to the stenoses. If the result is non-significant, it would be safe to defer revascularization and continue with optimal medical therapy (OMT) except in a patient where one of the lesions is critical which can cause a negative iFR/positive FFR pattern where evaluation should be continued with FFR. If the value is below 0.89, then FFR should be measured. If the result is above a threshold of 0.80, then revascularization should be deferred. On the other hand, if FFR < 0.80, then pullback should be done using hyperemia-induced FFR with a measurement of PPG. If there is no discrete step-up in FFR values during pullback and the PPG value is below 0.40, then there is a diffuse disease and (OMT) might provide a solution. If PPG is greater than 0.70, one should proceed with the intervention by treating the lesion that caused the greatest change in FFR value (*Δ*P) during pullback. Afterward, FFR measurement should be repeated, and if a significant FFR value persists, then PCI of the second lesion should be done, and measurements repeated after stent implantation and optimization (Figure 6).

## Conclusion

Serial stenosis evaluation remains a challenging issue in contemporary interventional cardiology. Invasive coronary physiology might provide a solution for the safe treatment of these demanding lesions, but it has to integrate stenosis evaluation by FFR or NHI and the diffuseness of the disease by PPG. Before gaining widespread acceptance, this strategy needs to be evaluated in a clinical trial that would compare it to a pullback NHI or FFR.
